# Breast cancer screening patterns and associated factors in Iranian women over 40 years

**DOI:** 10.1038/s41598-024-66342-0

**Published:** 2024-07-03

**Authors:** Elham Seyedkanani, Mina Hosseinzadeh, Mojgan Mirghafourvand, Leila Sheikhnezhad

**Affiliations:** 1 Department of Community Health Nursing, Nursing and Midwifery Faculty, Tabriz University of Medical Sciences, Tabriz, Iran; 2 Social Determinants of Health Research Center, Tabriz University of Medical Sciences, Tabriz, Iran

**Keywords:** Breast cancer, Screening, Prevention, Cancer, Health care

## Abstract

Screening is a key component of breast cancer early detection programs that can considerably reduce relevant mortality rates. The purpose of this study was to determine the breast cancer screening behavioral patterns and associated factors in women over 40 years of age. In this descriptive‑analytical cross‑sectional study, 372 over 40 years of age women visiting health centers in Tabriz, Iran, in 2023 were enrolled using cluster sampling. The data were collected using the sociodemographic characteristics questionnaire, breast cancer perception scale, health literacy for Iranian adults scale, and the Breast Cancer Screening Behavior Checklist. The obtained data were analyzed in SPSS version 16 using descriptive statistics (frequency, percentage, mean, and standard deviation) and inferential statistics (univariate and multivariate logistic regression analyses). In total, 68.3% of all participants performed breast self‑examination (BSE) (9.9% regularly, once per month), 60.2% underwent clinical breast examination (CBE) (8.9% regularly, twice per year), 51.3% underwent mammography (12.3% regularly, once per year), and 36.2% underwent sonography (3.8% regularly, twice per year). The findings also showed that women with benign breast diseases were more likely to undergo CBE (OR = 8.49; 95% CI 2.55 to 28.21; P < 0.001), mammography (OR = 8.84; 95% CI 2.98 to 10; P < 0.001), and sonography (OR = 18.84; 95% CI 6.40 to 53.33; P < 0.001) than others. Participants with low and moderate breast cancer perception scores were more likely to perform BSE than women with high breast cancer perception scores (OR = 2.20; 95% CI 1.21 to 4.00; P = 0.009) and women who had a history of benign breast disease were more likely to perform screening behaviors than others (OR = 2.47; 95% CI 1.27 to 4.80; P = 0.008). Women between the ages of 50 and 59 were more likely to undergo mammography (OR = 2.33; 95% CI 1.29 to 4.77; P = 0.008) and CBE (OR = 2.40; 95% CI 1.347 to 4.20; P = 0.003) than those ≥ 60 years. Given the low participation of women in regular breast cancer screening, it is suggested that health care providers highlight the need for screening at the specified intervals in their training programs. In addition, health authorities are recommended to use reminder systems to remind women, especially those over 40 years of age, of the best time for breast screening. Moreover, health care providers must seek to improve breast cancer knowledge, attitudes, and perceptions of women who visit health centers, which are the first level of contact with the healthcare system for the general population.

## Introduction

Breast cancer is one of the most serious health conditions worldwide, and the most common malignancy in women^1^. It is estimated that by the end of 2024, 310,720 new cases of breast cancer will be added to the number of women diagnosed with breast cancer in the United States, and 42,250 deaths will occur due to this disease^2^. In 2021, about 1.7 million new cases of breast cancer were identified worldwide, accounting for about 25% of all cancer cases in women^3^.

According to world health organization (WHO) reports, the number of breast cancer cases in the Middle East is expected to double by 2030^4^. In a 2017 National Cancer Registry Program, breast cancer was cited as the most common cancer in Iran and East Azerbaijan Province. In addition, the age-standardized incidence rate of breast cancer in Iran and East Azerbaijan was reported to be 43.02 and 40.72 (per 100,000 people), respectively^5^. In Iran, breast cancer occurs mainly in women between the ages of 45 and 55, indicating that Iranian women develop breast cancer a decade earlier than those in developed countries^6^. Therefore, breast cancer is considered a serious, life-threatening disease in women^7^.

Breast cancer is among the most preventable cancers^8^; lifestyle changes and early diagnosis can reduce the incidence and mortality rates of this cancer^9^. Primary prevention of cancer involves identifying relevant causative and risk factors and offering solutions to reduce these factors. Secondary prevention, on the other hand, includes timely screening and rapid treatment of cancer patients^10^. According to the guidelines of the Iranian Ministry of Health and Medical Education, there are three main methods for breast cancer screening, women over 20 years are recommended to perform breast self-examinations (BSE) monthly, while clinical breast examinations (CBE) by a healthcare professional are recommended annually for those over 40 years. Imaging techniques like mammograms and ultrasounds are also generally advised annually for women aged over 40^11^. A woman can perform BSE at any time and place monthly at no cost. The American Cancer Society (ACS) recommends that women over 40 years of age undergo mammography and CBE every year. In addition, women are recommended to have CBEs every 3 years between the ages of 20 and 40. Both mammography and CBE reduce breast cancer mortality rates by facilitating early detection and treatment^12^. Key goals of the Healthy People Initiative 2020–2030, launched by the U.S. Office of Disease Prevention and Health Promotion, include reducing breast cancer deaths, decreasing the number of late-stage breast cancer patients, and enhancing diagnostic behavior^7^.

Factors such as sociodemographic characteristics (e.g., age, educational qualifications, and income status), beliefs, and attitudes can affect women’s screening behavior^13^. Behavioral models and theories such as the Health Belief Model (HBM) highlight the crucial role of beliefs and cognitions in inspiring people to engage in healthy or risky behaviors (e.g., following or rejecting screening recommendations of physicians)^14^. According to the HBM, identifying patients’ negative beliefs and attitudes would help improve the effectiveness of training and treatments offered by health care providers^15^. Due to their poor health literacy, many women are unaware of the importance of cancer screening^16^. Health literacy refers to a person’s ability to receive, process, perceive, and understand health-related information in order to make appropriate health decisions. It is also an important factor that empowers women to take preventive measures to promote the health of themselves and their children ^17^. Research suggests that inadequate health literacy has negative consequences, particularly for cancer control, such as poor understanding of cancer risks, low perception of the importance of screening, and poor participation in preventing adverse clinical outcomes^18^. Low health literacy is generally associated with poor knowledge of cancer screening, unwillingness to undergo cancer examinations, limited access to treatment, improper use of medications, non-adherence to physician recommendations, an increase in hospitalization rates, and a heavy financial burden on the individual, family, and society^19^.

Considering the high prevalence of breast cancer in Iran and worldwide, its good prognosis, the tremendous importance of early diagnosis and screening, and the lack of studies on breast cancer screening patterns and related factors in Iran, this study investigated the screening patterns and associated factors in women over 40 years of age visiting health centers in Tabriz, Iran.

## Methods

### Study design and participants

In this descriptive-analytical cross-sectional study, 372 women over 40 years of age visiting health centers in Tabriz to receive various health services in 2022–2023 participated. Tabriz is one of the largest cities in northwestern Iran and the capital city of East Azerbaijan Province. With a population of over 1.7 million people, Tabriz has 10 municipal districts and 83 health centers.

The study included women over 40 years, regardless of marital status, who regularly visited health centers. Women with major, documented mental health conditions (such as major depressive disorder, bipolar disorder, Schizophrenia, or any other mental illness requiring ongoing medical treatment) in the SIB System or a history of breast cancer were excluded. The initial sample size was calculated as 171 based on the study of Taylan et al.^15^, considering the largest standard deviation of the domain of perceived fear, SD = 1.09, α = 0.05, d = 0.05, and mean = 3.27. The final sample size was determined as 342, taking into account a design effect of 2. However, because a large number of women visited the centers and the sampling process was very easy, 372 women were finally included in the study.

### Sampling

Participants were selected between November 2022 and March 2023 using cluster sampling. To this end, the researcher first randomly selected two health centers from each of the ten districts of Tabriz. After obtaining the list of eligible women from the “SIB System”, participants from each center were selected using proportional allocation and random numbers available on the “www.random.org” website. Then, the researcher screened the selected women by telephone for inclusion and exclusion criteria and briefly informed the eligible ones of the research objectives. The women were then asked to visit the respective health centers at a specific time to participate in the study. In the next step, an in-person session was held to explain the research objectives to all participants and to obtain informed consent from those who were willing to take part in the study. Finally, the researcher interviewed the participants and completed the research questionnaires.

### Data collection tools

The Sociodemographic Characteristics Questionnaire (SCQ), the Breast Cancer Perception Scale (BCPS), the Health Literacy for Iranian Adults (HELIA) Scale, and the Breast Cancer Screening Behavior Checklist were used to collect the data.

SCQ consisted of 15 items, including age, employment status, marital status, spouse’s job, spouse’s age, spouse’s education, and income sufficiency (the level of income that allows an individual or household to fully meet their basic needs and having acceptable standard of living), number of children, history of underlying diseases, history of breast cancer in family members, long-term use of hormonal medications (e.g., birth control pills), menopausal status, and self- or family history of benign breast disease.

BCPS was designed by Taylan et al. (2021) based on the HBM and its psychometric properties was assessed. The six domains of this 24-item scale include perceived knowledge (items 1–4), perceived treatment belief (items 5–9), perceived need for a health check (items 10–13), perceived stigma (items 14–17), perceived fear (items 18–21), and perceived risk (items 22–24). The items are scored on a five-point Likert scale from strongly disagree (1) to strongly agree (5), but items 9, 10, 11, 12, and 13 are scored inversely. Score range was between 24 and 120 and higher total scores indicate greater perception of breast cancer. Taylan et al. confirmed the construct and content validity of the instrument, and the reliability of all its domains was confirmed with Cronbach’s alpha values ranging from 0.81 to 0.95^15^.

The Health Literacy for Iranian Adults (HELIA) Scale developed by Montazeri et al. (2014) was used to measure the level of health literacy. The five subscales of this 33-item tool include reading (4 items), understanding (7 items), access (6 items), decision (12 items), and appraisal (4 items). A five-point Likert scale ranging from never (score 1) to always (score 5) is used to score the items. However, the items in the reading subscale are scored on a five-point Likert scale ranging from extremely difficult (score 1) to extremely easy (score 5). Score range was between 33 and 165 and higher score indicates higher health literacy. Montazeri et al. confirmed HELIA’s internal consistency reliability with a Cronbach’s alpha value of 0.88 and verified its content validity with a content validity index (CVI) and a content validity ratio (CVR) of 0.79 and 0.85, respectively in Iranian population^20^.In this study, the validity of HELIA scale was assessed only qualitatively using a survey of 10 faculty members of the Tabriz University Medical Sciences, and the scale items were not changed. We assessed the internal consistency of HELIA scale using Cronbach's alpha coefficient, which resulted in a value of 0.95.

The researcher designed a self-report 4-item checklist based on the guidelines of the Iranian Ministry of Health and Medical Education to examine participants’ breast cancer screening behaviors. This checklist was used by trained research staff to verbally assess participants' attendance at screening programs. The Persian version of this checklist is available as supplementary file.

Before starting the study, the face and content validity of the BCPS and the Breast Cancer Screening Behavior Checklist were assessed using quantitative and qualitative methods. In the qualitative phase, 10 faculty members of Tabriz University of Medical Sciences reviewed the Persian version of the questionnaires and provided corrective feedbacks on the use of correct vocabulary, grammar, etc. In the quantitative phase, CVR and CVI values were calculated. Based on Lawshe table, the minimum acceptable CVI and CVR values are 0.79 and 0.62, respectively. The CVI and CVR values for the BCPS were 0.98 and 0.95, respectively, whereas the values for the Breast Cancer Screening Behavior Checklist were 0.87 and 0.84, respectively. The reliability of BCPS and breast cancer screening behaviors checklist was assessed using the test–retest reliability. The intra-class correlation coefficient (ICC) for 30 individuals who completed the questionnaires twice at a two-week interval was calculated to be 0.97 for BCPS and 0.81 for breast cancer screening behaviors checklist. In addition, Cronbach’s alpha values of 0.68 was obtained for BCPS.

### Data analysis

The data were analyzed by SPSS version16. The normality of the quantitative data was first confirmed by assessing skewness and kurtosis and visual charts. Then, descriptive statistics of frequency (percentage) and mean (SD) were used to examine participants’ breast cancer perception, screening behavior, and health literacy levels. The binary logistic regression test was used to examine the relationship between screening behaviors and other research variables. Accordingly, multivariate logistic regression analysis was performed with backward strategy to adjust the socio-demographic variables. Here, each of the screening behaviors (BSE, CBE, mammography, and sonography) was considered separately as a dependent variable, and variables that had significant relationships with each of these variables (*p* < 0.2) were inserted as independent variables into a backward stepwise multivariate logistic regression analysis model. *P* value < 0.05 was considered significant.

### Ethics approval and consent to participate

The current study received approval from the Research Vice-Chancellor and the Ethics Committee at Tabriz University of Medical Sciences under the code IR.TBZMED.REC.1401.704. Initially, the objectives of the research, participant anonymity, voluntary involvement, and study details were verbally communicated. Subsequently, these were read and acknowledged through a signed written informed consent form. The research methodology adhered to the principles of the Helsinki Declaration.

## Results

The study sample consisted of 372 eligible women over 40 years of age visiting health centers in Tabriz. Abou half of participants (54.6%) were housewives. Most of them (79.6%) were married, and had sufficient income (covering expenses or more) (60.7%). In addition, 39 individuals (10.5%) and 74 individuals (19.9%) had a personal history and a family history of benign breast disease, respectively. Table 1 shows other sociodemographic characteristics of participants.

**Table 1:** Demographic characteristics of participants (N = 372).

Variable	Mean (SD*)	Characteristic	Number (percent)
One’s own age	52.46 (8.94)	History of disease	
Husband age	56.70 (9.04)	Yes	161 (43.3)
Number of children	2.32 (1.35)	No	211 (56.7)
Characteristic	Number (percent)	History of breast cancer	
Income status		Yes	49 (13.2)
Income more than expenses	57 (15.3)	No	323 (86.4)
Income equal to expenses	169 (45.4)	History of taking hormonal drugs	
Income less than expenses	146 (39.2)	Yes	88 (23.7)
Education		No	284 (76.3)
Illiterate	34 (9.1)	History of benign breast disease	
Elementary-middle school	89 (23.9)	Yes	39 (10.5)
High school, diploma	96 (25.8)	No	333 (89.5)
Higher diploma	17 (4.6)	History of benign breast disease in the relatives	
Bachelor’s degree	106 (28.5)	Yes	74(19.9)
Master’s degree	26(7)	No	298(80.1)
Ph.D	4(1.1)	Menopause	
Husband education		Yes	205(55.1)
Illiterate	16 (4.3)	No	167(44.9)
Elementary-middle school	77 (20.7)	Marital status	
High school, diploma	68 (18.3)	Single	33(8.9
Higher diploma	17 (4.6)	Married	296(79.6)
Bachelor’s degree	75 (20.2)	Divorced/widowed	43(11.6)
Ph.D	15(4.0)	Breast cancer perception	
The individual's employment status		Low	7(1.9)
Household worker	24 (6.5)	Average	307 (82.5)
Employed	99 (26.6)	Satisfactory	85 (15.6)
Housewife	203 (54.6)		
Retired	46 (12.4)		
Husband's employment			
Employee	60 (16. 1)		
Worker	22 (5.9)		
Self-employment	111 (29.8)		
Managerial role	12 (3.2)		
Retired	91 (24.5)		

Overall, 68.3% of all women had performed BSE at least once, but only 9.9% of them performed regular monthly examinations. In addition, 60.2% of women underwent CBE at least once, but only 8.9% of them underwent regular CBE every 6 months. Moreover, 51.3% of participants underwent mammography at least once, but only 12.3% of them attended regular annual mammography sessions. Finally, 38.2% of women underwent sonography at least once, but only 3.8% of them had regular sonography every 6 months (Table 2).

**Table 2:** Frequency of breast cancer screening behaviors in women (N = 372).

Variable		Frequency	Percentage	Variable		Frequency	Percentage
Breast self-examination	Yes	254	68.3	Mammography	Yes	191	51.3
	No	118	31.7		No	181	48.7
Execution intervals				Execution intervals			
Every month		37	9.9	Every year		48	12.9
Every 2 months		21	5.6	Every 2 years		27	7.3
Every 2 to 6 months		19	5.1	Every 2 to 5 years		29	7.8
Every 6 to 12 months		79	21.2	More than 5 years apart		29	7.8
Irregular		99	26.6	Irregular		58	15.6
Examination by physician	Yes	224	60.2	Ultrasound	Yes	142	38.2
	No	148	39.8		No	230	61.8
Execution intervals				Execution intervals			
Every 6 months		33	8.9	Every 6 months		14	3.8
Every year		54	14.5	Every year		25	6.7
Every 2 years		18	4.8	Every 2 years		22	5.9
More than 2 years apart		38	10.2	Every 2 to 5 years		25	6.7
Irregular		81	21.8	More than 5 years apart		17	4.6
				Irregular		41	11

The univariate logistic regression analysis results showed that variables of age, educational qualifications, spouse’s age, spouse’s educational qualifications, spouse’s job, history of underlying diseases, history of breast cancer in family members, self or family history of benign breast disease, breast cancer perception, and health literacy had significant relationships with BSE (*p* < 0.2). Therefore, these variables were entered into a backward stepwise multivariate analysis model. The results showed that both family history of benign breast disease (OR = 2.47; 95% CI 1.27 to 4.80; *P* = 0.008) and breast cancer perception (OR = 2.20; 95% CI 1.21 to 4.00; *P* = 0.009) were significantly associated with BSE. In other words, women with a family history of benign breast disease were 2.47 times more likely to perform BSE than others. In addition, women with low and moderate breast cancer perception scores were 2.2 times more likely to perform BSE than those with high perception scores (Table 3).

**Table 3:** Related factors of breast cancer screening behaviors according to multivariate logistic regression model (N = 372).

*BSE*		
Variables	aOR* (95% CI)	*P* value
Education (Reference: University degree)		
Diploma and lower	1.54 (0.96–2.46)	0.068
History of benign breast disease (Reference: NO)		
Yes	1.88 (0.77–4.55)	0.160
History of benign breast disease in the relatives (Reference: No)		
Yes	2.47 (1.27–4.80)	0.008
Breast cancer perception (Reference: Satisfactory)		
Low and average	2.20 (1.21–4.00)	0.009
*Examination by physician*		
Age (Year) (Reference: 60 and more)		
40–49	1.59 (0.91–2.75)	0.102
50–59	2.40 (1.34–4.29)	0.003
History of benign breast disease (Reference: NO)		
Yes	8.49 (2.55–28.21)	< 0.001
*Mammography*		
Age (Year) (Reference: 60 and)		
40–49	1.15 (0.65–1.53)	0.618
50–59	2.33 (1.29–4.77)	0.005
History of benign breast disease (Reference: NO)		
Yes	8.84 (2.98–10.00)	< 0.001
History of benign breast disease in the relatives (Reference: No)		
Yes	1.77 (0.99–1.00)	0.054
Breast cancer perception (Reference: satisfactory)		
Low and average	1.83 (0.99–1.00)	0.052
*Ultrasound as a complement to mammography*		
Benign breast disease (Reference: NO)		
Yes	18.84 (6.40–53.33)	0 < 001

*Adjusted Odd ratio.

The univariate logistic regression analysis results showed that variables of age, number of children, spouse’s age, self- or family history of benign breast disease, breast cancer perception, and health literacy had significant relationships with CBE (*p* < 0.2). Therefore, these variables were entered into a backward stepwise multivariate analysis model. Based on the results, variables of age (OR = 2.40; 95% CI 1.347 to 4.20; *P* = 0.003) and personal history of benign breast disease (OR = 8.49; 95% CI 2.55 to 28.21; *P* < 0.001) were significantly related to CBE. In other words, women who had a history of benign breast disease were 8 times more likely to undergo CBE than others. In addition, women between the ages of 50 and 59 were 2.4 times more likely to undergo CBE than those over 60 years (Table 3).

The univariate logistic regression analysis results showed that variables of age, employment status, spouse’s age, income status, history of underlying diseases, history of breast cancer in family members, history of use of hormonal medications, self or family history of benign breast disease, and breast cancer perception had significant relationships with mammography screening (*p* < 0.2). These variables were entered into a backward stepwise multivariate analysis model. The results indicated that variables of age (OR = 2.33; 95% CI 1.29 to 4.77; *P* = 0.008) and personal history of benign breast disease (OR = 8.84; 95% CI 2.98 to 10; *P* < 0.001) were significantly associated with mammography screening. This implied that women who had a history of benign breast disease were 8.8 times more likely to undergo mammography than others. In addition, women between the ages of 50 and 59 were 2.38 times more likely to undergo mammography than those over 60 years (Table 3).

Finally, the univariate logistic regression analysis results showed that variables of educational qualifications, spouse’s age, spouse’s educational qualifications, number of children, income status, history of underlying diseases, history of breast cancer in family members, self- or family history of benign breast disease, and breast cancer perception had significant relationships with sonography screening (*p* < 0.2). After inserting these variables into a backward stepwise multivariate analysis model, the results showed that variable of personal history of benign breast disease (OR = 18.84; 95% CI 6.40 to 53.33; *P* < 0.001) was significantly associated with sonography screening. In other words, women who had a history of benign breast disease were 18.48 times more likely to undergo sonography than others (Table 3).

## Discussion

This study aimed to determine the breast cancer screening behaviors patterns and associated factors among women over 40 years of age. Based on the findings, more than half of participants experienced BSE, CBE, and mammography at least once; however, few performed these examinations regularly and according to recommended guidelines. A history of benign breast disease was significantly associated with all four screening behaviors, as women who had a history of benign breast disease were more likely to perform screening behaviors than others. Women with low and moderate breast cancer perception scores were more likely to perform BSE than those with high breast cancer perception scores. In addition, women between the ages of 50 and 59 were more likely to undergo mammography and CBE than those ≥ 60 years.

Our study found that while 68.3% of participants reported performing BSE at least once, only 9.9% performed it regularly each month. This highlights a gap in adherence, similar to findings from other studies. For instance, Kwok (2020) observed that in Korean-Australian women, despite awareness of breast cancer screening methods (including BSE), only 31.4% performed regular BSE^21^. Similarly, a study in Korea reported high awareness of BSE benefits (88%) but low practice (29.3%), with many participants citing lack of proper knowledge (31.7%) as a barrier^22^. Likewise, another study in Istanbul, Turkey, found only 32.1% of women performing regular BSE^23^.

The prevalence of regular BSE in this study was about 10% which is lower than in similar studies. Unfamiliarity with recommended intervals might be a contributing factor. Healthcare providers should emphasize the importance of regular BSE practice. Currently, there is continuous debate about the effectiveness of BSE and CBE in reducing mortality rates. As a result, some international organizations no longer recommend these examinations as screening measures for detecting breast cancer. However, in less developed countries, where women are often diagnosed with breast cancer at a younger age and at advanced stages, the advantages of these methods may outweigh their disadvantages and facilitate early detection of breast cancer^24^. The results of a recent 5 year follow-up study showed that women who perform BSE irregularly have a 1.31 times higher risk of developing late-stage breast cancer and a 1.70 times higher risk of dying from breast cancer than those who perform it regularly. In addition, women who had regular BSE screening had significantly smaller tumors, earlier cancer stage, and higher survival rates than others. In developing countries, women between the ages of 50 and 74 are usually recommended to have regular mammograms every 2 or 3 years. However, since mammography services are not extensively provided to younger women, BSE is still widely used in these countries^25^.

Similar to BSE, CBE adherence was low in our study, with only 8.9% of women receiving regular screenings. In a similar study in Iran by Rabiei et al. (2022), it was reported a 52.6% prevalence of ever-performed CBE in Iranian women, but did not specify regular screening rates ^26^. The prevalence of CBE in this study (60.2%) was higher than in the study of Rabiei et al. (52.6%). A study in Malaysia in 2010 also found a lower prevalence (25%) of ever-performed CBE among female teachers^27^. The difference is probably due to the older age of the participants in this study compared to similar studies. Accordingly, recent studies have found a direct association between age and the prevalence of CBE. CBE performed by health care providers at recommended intervals significantly lowers the stage of cancer diagnosis and reduces mortality by 15% in women. CBE also results in a 30% reduction in mortality rates in women older than 50 years^28^. In contrast, the findings of a study on 11 systematic reviews showed no direct evidence that CBE reduces mortality in breast cancer patients; however, CBE reduced the likelihood of shifting from early-stage to advanced-stage cancer by 17–47%^29^.

This study found that while over half (51.3%) of participants underwent mammography at least once, only 12.3% adhered to recommended screening intervals. This low adherence rate aligns with findings from developing countries^21,30,31^, where prevalence of screening is generally lower compared to developed nations (e.g., 70% in the United States)^32^. This suggests that interventions beyond awareness campaigns might be needed in developing countries. Furthermore, research in developed nations, such as the United States^32^, has identified physician recommendation as a key factor influencing screening behavior. This suggests a valuable direction for future research in developing countries. Regarding that in the present study only a small percentage of women had mammograms at the recommended intervals, regularly visits for older women by doctors at the first level of the healthcare system and advising them to have regular mammograms is suggested.

Self or family history of benign breast disease was significantly associated with all four screening behaviors. In line with this finding, studies report that a family history of cancer^33,34^ and a history of breast diseases in oneself^35^, friends, and peers^36^ have significant positive relationships with breast cancer screening behaviors such as CBE and mammography. In addition, perceived cancer risk is higher in these women than in others, and according to the HBM, high perceived risk is related to a high likelihood of engaging in preventive behaviors. In a recent study, 70% of participants reported family history as the main risk factor for developing breast cancer. For this reason, women with a family history of benign breast disease are more inclined than others to participate in breast cancer screening^37^. In this study, women with a family history of benign breast disease were more likely to perform BSE than others. In contrast to the present results, Guo observed no consistent relationship between family history of breast lumps and screening behavior (mammography and CBE)^38^. The discrepancy between Guo’s findings and the present results can be attributed to the fact that some women experience psychological problems such as fear, and therefore exhibit avoidance behaviors. Regardless of personal or family history, regular mammograms remain crucial for all women within the recommended age range. Therefore, future public health initiatives should prioritize messaging that underscores the importance of regular screening for all women within the recommended age range, irrespective of perceived risk.

This study found a positive association between age and both CBE and mammography utilization. This aligns with some existing research, where older women were reported to be more likely to undergo screening^36^. However, conflicting evidence also exists^39^. Consistent with the findings of this study, a 2018 systematic review in Malaysia reported age as an important predictor of mammography because older women were more likely to seek mammography^40^. These discrepancies might be due to factors like cost and insurance coverage, as suggested in studies where younger women had limited access to mammograms due to insurance policies^39^. For example, some health insurance companies may not cover screening mammograms for women younger than 40 years^41^. This highlights the potential influence of socioeconomic factors on screening behavior. It's important to note that some studies report a decrease in screening adherence among women over 65 years old^42^. This suggests a more nuanced relationship between age and screening behavior, where factors like health status might play a role in later life. Additionally, research suggests that interventions like increasing awareness campaigns, reminder systems, and offering subsidized or free screening services, particularly for older women, can be effective in improving screening rates^43^.

This study found an intriguing relationship between breast cancer perception and BSE behavior. Women with lower and moderate perceptions were more likely to perform BSE compared to those with high perception. While the reasons for this require further investigation, existing research suggests a connection between breast cancer awareness, knowledge, and health-seeking behaviors^44^. It's possible that women with lower perceptions might have low perceived need for a health check, poor perceived treatment beliefs, and great perceived fear of breast cancer leading them to rely on self-examinations (BSE) as a form of control or reassurance. Rainey et al. (2019) examined women’s perceptions of breast cancer screening in three countries and concluded that women’s perceptions of screening, which are rooted in behavioral theory, are influenced by factors such as lack of knowledge, cultural norms, and common emotional concerns^45^. Therefore, providing appropriate educational materials and risk counseling programs can help women make informed collective or individual decisions. It should be noted that acceptance of risk-based screening and prevention of breast cancer are intertwined^21^. Accordingly, appropriate educational programs should be provided at the national level to institutionalize screening behaviors among women by improving their perception and raising their awareness of breast cancer. Nurses can play a vital role in raising breast cancer awareness among women by organizing comprehensive screening programs^46^.

## Strengths and limitations

In this study, the factors associated with each of the four recommended screening behaviors were examined separately that can consider as a strength of our study. The second strength of the study was the random selection of participants among all health centers in Tabriz. However, our study has some limitations. Use of self-report questionnaires increased the risk of bias in this study. Moreover, due to the cross-sectional nature of the study, the relationships among the research variables cannot be considered as cause-and-effect relationships. Our reliance on a selected cohort of consenting women limits the generalizability of the findings to the broader Iranian population. A broader retrospective analysis across the healthcare system could mitigate this limitation.

## Conclusion

Given the low participation of women in regular breast cancer screening, it is suggested that health care providers emphasize the need for screening at the specified intervals in their training programs. Considering the high prevalence of breast cancer in Iran, relevant health authorities are recommended to use reminder systems to remind Iranian women, especially those over 40 years of age, of the best time for breast screening. Moreover, health care providers must seek to improve breast cancer knowledge, attitudes, and perceptions of women who visit health centers, which are the first level of contact with the healthcare system for the general population.

### Supplementary Information

Supplementary Information.

## Data Availability

The datasets used and/or analyzed in the current study are available through the corresponding author for scientific use, such as replication.
