# Supplementary material for: Breast cancer screening patterns and associated factors in Iranian women over 40 years
Source: Sci Rep. 2024 Jul 3;14:15274. doi: 10.1038/s41598-024-66342-0 (PMC11222508; doi:10.1038/s41598-024-66342-0)
Supplement: Supplementary file 1 — Supplementary Information. [file 41598_2024_66342_MOESM1_ESM.docx]

**Supplementary file: Breast cancer screening behaviors checklist**

**Dear contributor, this checklist is designed to examine breast cancer screening behaviors. Please help us reach the goal of the research by answering the questions accurately.**

1. Have you ever had a breast examination? Yes  No 

If your answer is yes, how often do you do the examination?

Every month  Every 2 months  Every 2 to 6 months  Every 6 to 12 months  Irregularly and less than 2 times a year 

1. Has a doctor, health care provider or midwife ever examined your breast?

Yes  No

If your answer is yes, how often was this examination done?

Every six months  Once every year  Once every two years  At intervals of more than two years 

1. Have you ever had a mammogram? Yes  No

If your answer is yes, how often have you done mammography?

Once every year  Once every two years  At intervals of 2 to 5 years  At intervals of more than five years 

1. Have you done a breast ultrasound (as a supplement to mammography)? Yes  No

If yes, how often have you done this?

Every six months  Once every year  Once every two years  At intervals of 2 to 5 years  At intervals of more than five years 
